# 4,4′-Dibromo-2,2′-[octane-1,8-diylbis(nitrilo­methanylyl­idene)]diphenol

**DOI:** 10.1107/S1600536811030790

**Published:** 2011-08-06

**Authors:** Kwang Ha

**Affiliations:** a School of Applied Chemical Engineering, The Research Institute of Catalysis, Chonnam National University, Gwangju 500-757, Republic of Korea

## Abstract

The title compound, C_22_H_26_Br_2_N_2_O_2_, has a centre of inversion that is located in the middle of the octyl chain; the chain displays an extended zigzag conformation. A short intra­molecular O—H⋯N hydrogen bond occurs.

## Related literature

For related structures, see: Elerman *et al.* (1998 ▶); Ünaleroğlu & Hökelek (2002 ▶).
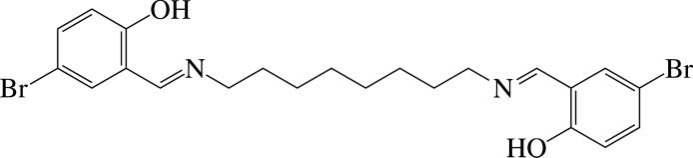


## Experimental

### 

#### Crystal data

C_22_H_26_Br_2_N_2_O_2_
*M* *_r_* = 510.27
Triclinic, 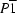

*a* = 8.253 (3) Å
*b* = 8.363 (3) Å
*c* = 9.571 (3) Å
α = 64.431 (6)°
β = 65.839 (7)°
γ = 87.403 (7)°
*V* = 536.7 (3) Å^3^
*Z* = 1
Mo *K*α radiation
μ = 3.80 mm^−1^
*T* = 200 K
0.24 × 0.23 × 0.10 mm

#### Data collection

Bruker SMART 1000 CCD diffractometer
Absorption correction: multi-scan (*SADABS*; Bruker, 2000 ▶) *T*_min_ = 0.701, *T*_max_ = 1.000
3910 measured reflections
2557 independent reflections
1445 reflections with *I* > 2σ(*I*)
*R*_int_ = 0.046

#### Refinement

*R*[*F* ^2^ > 2σ(*F* ^2^)] = 0.066
*wR*(*F* ^2^) = 0.176
*S* = 1.04
2557 reflections
130 parameters
H atoms treated by a mixture of independent and constrained refinement
Δρ_max_ = 0.69 e Å^−3^
Δρ_min_ = −0.67 e Å^−3^

### 

Data collection: *SMART* (Bruker, 2000 ▶); cell refinement: *SAINT* (Bruker, 2000 ▶); data reduction: *SAINT*; program(s) used to solve structure: *SHELXS97* (Sheldrick, 2008 ▶); program(s) used to refine structure: *SHELXL97* (Sheldrick, 2008 ▶); molecular graphics: *ORTEP-3* (Farrugia, 1997 ▶) and *PLATON* (Spek, 2009 ▶); software used to prepare material for publication: *SHELXL97*.

## Supplementary Material

Crystal structure: contains datablock(s) global, I. DOI: 10.1107/S1600536811030790/ng5205sup1.cif

Structure factors: contains datablock(s) I. DOI: 10.1107/S1600536811030790/ng5205Isup2.hkl

Supplementary material file. DOI: 10.1107/S1600536811030790/ng5205Isup3.cml

Additional supplementary materials:  crystallographic information; 3D view; checkCIF report

## Figures and Tables

**Table 1:** Hydrogen-bond geometry (Å, °)

*D*—H⋯*A*	*D*—H	H⋯*A*	*D*⋯*A*	*D*—H⋯*A*
O1—H1⋯N1	0.84 (7)	1.86 (7)	2.581 (7)	144 (7)

**Fig. 1.**
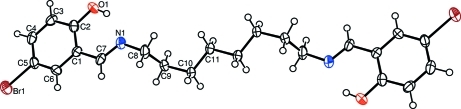
The structure of the title compound, with displacement ellipsoids drawn at the 50% probability level; H atoms are shown as small circles of arbitrary radius. Unlabelled atoms are related to the reference atoms by the (1 - x, 2 - y, -1 - z) symmetry transformation.

**Fig. 2.**
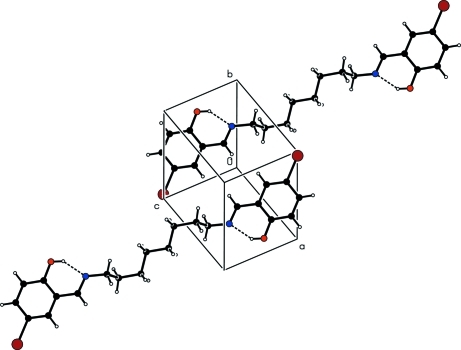
View of the unit-cell contents of the title compound. Hydrogen-bond interactions are drawn with dashed lines.

## supplementary crystallographic information

### Comment

The title compound, C_22_H_26_Br_2_N_2_O_2_, can act as a dibasic tetradentate ligand, that is, the N_2_O_2_ donor atoms can coordinate one or two metal ions (Fig. 1). The compound crystallized in the triclinic space group *P*1, whereas the related Schiff base with ethylene group (C_16_H_14_Br_2_N_2_O_2_) (Ünaleroğlu & Hökelek, 2002) and propylene chain (C_17_H_16_Br_2_N_2_O_2_) (Elerman *et al.*, 1998) crystallized in the monoclinic space groups *P*2_1_/*a* and *P*2_1_/*n*, respectively.

A centre of inversion is located at the centroid of the title molecule, and therefore the asymmetric unit contains one half of the formula unit and the two benzene rings are exactly parallel. The N1—C7/8 bond lengths and the C7—N1—C8 bond angle indicate that the imino N1 atom is *sp*^2^-hybridized [*d*(N1═C7) = 1.290 (7) Å and d(N1—C8) = 1.459 (7) Å; <C7—N1—C8 = 118.0 (5)°]. The C8—C9—C10—C11 torsion angle of -77.2 (7)° displays the *gauche* conformation for the four atoms within the diiminooctylene chain, whereas the N1—C8—C9—C10 and C9—C10—C11—C11^i^ (symmetry code i: 1 - *x*, 2 - *y*, -1 - *z*) atoms show the anti conformation with the torsion angle of 174.6 (5)° and -178.9 (6)°, respectively. The molecule reveals strong intramolecular O—H···N hydrogen bonding between the hydroxy O atom and the imino N atom with d(O···N) = 2.581 (7) Å forming a nearly planar six-membered ring (Fig. 2, Table 1).

### Experimental

1,8-Diaminooctane (1.0103 g, 7.003 mmol) and 5-bromosalicylaldehyde (2.8159 g, 14.008 mmol) in EtOH (20 ml) were stirred for 1 h at room temperature. After addition of pentane (30 ml) to the reaction mixture, the formed precipitate was separated by filtration, washed with ether, and dried at 50 °C, to give a yellow powder (2.8918 g). Crystals suitable for X-ray analysis were obtained by slow evaporation from a CH_3_CN solution.

### Refinement

H atoms were positioned geometrically and allowed to ride on their respective parent atoms [C—H = 0.95 Å (CH) or 0.99 Å (CH_2_) and *U*_iso_(H) = 1.2*U*_eq_(C)]. The hydroxy H atom was located from Fourier difference maps and refined isotropically with *U*_iso_(H) = 1.5*U*_eq_(O) [O—H = 0.84 (7) Å].

### Crystal data

**Table:**

C_22_H_26_Br_2_N_2_O_2_	*Z* = 1
*M**_r_* = 510.27	*F*(000) = 258
Triclinic, *P*1	*D*_x_ = 1.579 Mg m^−^^3^
Hall symbol: -P 1	Mo *K*α radiation, λ = 0.71073 Å
*a* = 8.253 (3) Å	Cell parameters from 1213 reflections
*b* = 8.363 (3) Å	θ = 2.7–28.0°
*c* = 9.571 (3) Å	µ = 3.80 mm^−^^1^
α = 64.431 (6)°	*T* = 200 K
β = 65.839 (7)°	Block, yellow
γ = 87.403 (7)°	0.24 × 0.23 × 0.10 mm
*V* = 536.7 (3) Å^3^	

### Data collection

**Table:**

Bruker SMART 1000 CCD diffractometer	2557 independent reflections
Radiation source: fine-focus sealed tube	1445 reflections with *I* > 2σ(*I*)
graphite	*R*_int_ = 0.046
φ and ω scans	θ_max_ = 28.3°, θ_min_ = 2.6°
Absorption correction: multi-scan (*SADABS*; Bruker, 2000)	*h* = −9→10
*T*_min_ = 0.701, *T*_max_ = 1.000	*k* = −10→11
3910 measured reflections	*l* = −10→12

### Refinement

**Table:**

Refinement on *F*^2^	Primary atom site location: structure-invariant direct methods
Least-squares matrix: full	Secondary atom site location: difference Fourier map
*R*[*F*^2^ > 2σ(*F*^2^)] = 0.066	Hydrogen site location: inferred from neighbouring sites
*wR*(*F*^2^) = 0.176	H atoms treated by a mixture of independent and constrained refinement
*S* = 1.04	*w* = 1/[σ^2^(*F*_o_^2^) + (0.0693*P*)^2^] where *P* = (*F*_o_^2^ + 2*F*_c_^2^)/3
2557 reflections	(Δ/σ)_max_ < 0.001
130 parameters	Δρ_max_ = 0.69 e Å^−^^3^
0 restraints	Δρ_min_ = −0.67 e Å^−^^3^

### Special details

**Table:**

Geometry. All e.s.d.'s (except the e.s.d. in the dihedral angle between two l.s. planes) are estimated using the full covariance matrix. The cell e.s.d.'s are taken into account individually in the estimation of e.s.d.'s in distances, angles and torsion angles; correlations between e.s.d.'s in cell parameters are only used when they are defined by crystal symmetry. An approximate (isotropic) treatment of cell e.s.d.'s is used for estimating e.s.d.'s involving l.s. planes.
Refinement. Refinement of *F*^2^ against ALL reflections. The weighted *R*-factor *wR* and goodness of fit *S* are based on *F*^2^, conventional *R*-factors *R* are based on *F*, with *F* set to zero for negative *F*^2^. The threshold expression of *F*^2^ > σ(*F*^2^) is used only for calculating *R*-factors(gt) *etc*. and is not relevant to the choice of reflections for refinement. *R*-factors based on *F*^2^ are statistically about twice as large as those based on *F*, and *R*- factors based on ALL data will be even larger.

### Fractional atomic coordinates and isotropic or equivalent isotropic displacement parameters (Å^2^)

**Table:**

	*x*	*y*	*z*	*U*_iso_*/*U*_eq_	
Br1	−0.15293 (11)	−0.11794 (9)	0.87746 (8)	0.0532 (3)	
O1	−0.2341 (6)	0.5706 (6)	0.3510 (5)	0.0402 (12)	
H1	−0.139 (10)	0.622 (10)	0.262 (9)	0.060*	
N1	0.1031 (7)	0.6095 (6)	0.1542 (6)	0.0350 (12)	
C1	−0.0431 (8)	0.3603 (8)	0.4327 (7)	0.0279 (13)	
C2	−0.2133 (8)	0.4146 (8)	0.4675 (7)	0.0308 (13)	
C3	−0.3617 (8)	0.3092 (8)	0.6202 (7)	0.0351 (15)	
H3	−0.4757	0.3477	0.6419	0.042*	
C4	−0.3464 (8)	0.1514 (8)	0.7395 (7)	0.0334 (14)	
H4	−0.4488	0.0792	0.8427	0.040*	
C5	−0.1777 (9)	0.0982 (8)	0.7068 (7)	0.0334 (14)	
C6	−0.0262 (8)	0.1996 (7)	0.5568 (7)	0.0336 (15)	
H6	0.0876	0.1611	0.5380	0.040*	
C7	0.1174 (8)	0.4680 (8)	0.2747 (7)	0.0295 (13)	
H7	0.2322	0.4338	0.2609	0.035*	
C8	0.2663 (9)	0.7103 (8)	−0.0021 (7)	0.0390 (16)	
H8A	0.3705	0.6960	0.0261	0.047*	
H8B	0.2560	0.8395	−0.0495	0.047*	
C9	0.2982 (9)	0.6468 (8)	−0.1364 (6)	0.0342 (15)	
H9A	0.1890	0.6500	−0.1560	0.041*	
H9B	0.3198	0.5209	−0.0929	0.041*	
C10	0.4597 (8)	0.7637 (7)	−0.3076 (6)	0.0331 (15)	
H10A	0.5599	0.7859	−0.2834	0.040*	
H10B	0.5002	0.6963	−0.3761	0.040*	
C11	0.4197 (8)	0.9434 (7)	−0.4143 (6)	0.0311 (14)	
H11A	0.3817	1.0120	−0.3470	0.037*	
H11B	0.3180	0.9215	−0.4368	0.037*	

### Atomic displacement parameters (Å^2^)

**Table:**

	*U*^11^	*U*^22^	*U*^33^	*U*^12^	*U*^13^	*U*^23^
Br1	0.0653 (6)	0.0326 (4)	0.0349 (4)	0.0100 (3)	−0.0125 (3)	−0.0017 (3)
O1	0.044 (3)	0.034 (2)	0.032 (2)	0.016 (2)	−0.017 (2)	−0.0065 (19)
N1	0.037 (3)	0.030 (3)	0.024 (2)	0.002 (2)	−0.008 (2)	−0.006 (2)
C1	0.022 (3)	0.030 (3)	0.024 (3)	0.003 (2)	−0.005 (2)	−0.011 (2)
C2	0.031 (4)	0.028 (3)	0.024 (3)	−0.004 (3)	−0.008 (2)	−0.006 (2)
C3	0.024 (4)	0.043 (4)	0.032 (3)	0.007 (3)	−0.006 (3)	−0.018 (3)
C4	0.036 (4)	0.032 (3)	0.022 (3)	−0.005 (3)	−0.004 (3)	−0.010 (2)
C5	0.042 (4)	0.027 (3)	0.021 (3)	0.003 (3)	−0.011 (3)	−0.005 (2)
C6	0.036 (4)	0.023 (3)	0.029 (3)	0.008 (3)	−0.010 (3)	−0.006 (2)
C7	0.026 (3)	0.031 (3)	0.023 (3)	−0.003 (3)	−0.001 (2)	−0.012 (2)
C8	0.041 (4)	0.035 (3)	0.021 (3)	−0.002 (3)	−0.005 (3)	−0.004 (3)
C9	0.038 (4)	0.026 (3)	0.022 (3)	0.005 (3)	−0.008 (3)	−0.002 (2)
C10	0.035 (4)	0.028 (3)	0.019 (3)	0.007 (3)	−0.004 (3)	−0.003 (2)
C11	0.027 (4)	0.028 (3)	0.022 (3)	0.001 (3)	−0.003 (2)	−0.004 (2)

### Geometric parameters (Å, °)

**Table:**

Br1—C5	1.915 (6)	C6—H6	0.9500
O1—C2	1.362 (7)	C7—H7	0.9500
O1—H1	0.84 (7)	C8—C9	1.518 (8)
N1—C7	1.290 (7)	C8—H8A	0.9900
N1—C8	1.459 (7)	C8—H8B	0.9900
C1—C6	1.406 (7)	C9—C10	1.538 (7)
C1—C2	1.407 (8)	C9—H9A	0.9900
C1—C7	1.465 (7)	C9—H9B	0.9900
C2—C3	1.390 (8)	C10—C11	1.522 (7)
C3—C4	1.365 (8)	C10—H10A	0.9900
C3—H3	0.9500	C10—H10B	0.9900
C4—C5	1.394 (8)	C11—C11^i^	1.530 (10)
C4—H4	0.9500	C11—H11A	0.9900
C5—C6	1.386 (8)	C11—H11B	0.9900
			
C2—O1—H1	113 (5)	N1—C8—H8A	109.3
C7—N1—C8	118.0 (5)	C9—C8—H8A	109.3
C6—C1—C2	118.6 (5)	N1—C8—H8B	109.3
C6—C1—C7	119.0 (5)	C9—C8—H8B	109.3
C2—C1—C7	122.3 (5)	H8A—C8—H8B	108.0
O1—C2—C3	119.5 (5)	C8—C9—C10	112.0 (5)
O1—C2—C1	120.1 (5)	C8—C9—H9A	109.2
C3—C2—C1	120.4 (5)	C10—C9—H9A	109.2
C4—C3—C2	121.2 (6)	C8—C9—H9B	109.2
C4—C3—H3	119.4	C10—C9—H9B	109.2
C2—C3—H3	119.4	H9A—C9—H9B	107.9
C3—C4—C5	118.7 (5)	C11—C10—C9	113.9 (5)
C3—C4—H4	120.7	C11—C10—H10A	108.8
C5—C4—H4	120.7	C9—C10—H10A	108.8
C6—C5—C4	122.0 (5)	C11—C10—H10B	108.8
C6—C5—Br1	118.9 (5)	C9—C10—H10B	108.8
C4—C5—Br1	119.0 (4)	H10A—C10—H10B	107.7
C5—C6—C1	119.1 (5)	C10—C11—C11^i^	113.3 (6)
C5—C6—H6	120.4	C10—C11—H11A	108.9
C1—C6—H6	120.4	C11^i^—C11—H11A	108.9
N1—C7—C1	120.0 (5)	C10—C11—H11B	108.9
N1—C7—H7	120.0	C11^i^—C11—H11B	108.9
C1—C7—H7	120.0	H11A—C11—H11B	107.7
N1—C8—C9	111.5 (5)		
			
C6—C1—C2—O1	179.2 (5)	Br1—C5—C6—C1	−178.7 (4)
C7—C1—C2—O1	1.4 (8)	C2—C1—C6—C5	1.6 (9)
C6—C1—C2—C3	−1.3 (9)	C7—C1—C6—C5	179.5 (5)
C7—C1—C2—C3	−179.2 (6)	C8—N1—C7—C1	−178.8 (5)
O1—C2—C3—C4	179.5 (5)	C6—C1—C7—N1	175.4 (5)
C1—C2—C3—C4	0.0 (9)	C2—C1—C7—N1	−6.8 (8)
C2—C3—C4—C5	0.9 (9)	C7—N1—C8—C9	91.7 (7)
C3—C4—C5—C6	−0.6 (9)	N1—C8—C9—C10	174.6 (5)
C3—C4—C5—Br1	177.4 (4)	C8—C9—C10—C11	−77.2 (7)
C4—C5—C6—C1	−0.7 (9)	C9—C10—C11—C11^i^	−178.9 (6)

Symmetry codes: (i) −*x*+1, −*y*+2, −*z*−1.

### Hydrogen-bond geometry (Å, °)

**Table:**

*D*—H···*A*	*D*—H	H···*A*	*D*···*A*	*D*—H···*A*
O1—H1···N1	0.84 (7)	1.86 (7)	2.581 (7)	144 (7)
